# Household Willingness-to-Pay for Improved Solid Waste Management Services in Four Major Metropolitan Cities in Ghana

**DOI:** 10.1155/2019/5468381

**Published:** 2019-01-02

**Authors:** Kofi Sekyere Boateng, Peter Agyei-Baffour, Daniel Boateng, George Nana Kwasi Rockson, Kofi Akohene Mensah, Anthony Kwaku Edusei

**Affiliations:** ^1^School of Public Health, Kwame Nkrumah University of Science and Technology, Kumasi, Ghana; ^2^African Institute of Sanitation and Waste Management, Kwame Nkrumah University of Science and Technology, Accra, Ghana

## Abstract

**Introduction:**

Waste management is an important developmental issue globally, especially in developing countries like Ghana. A key challenge of waste management in developing countries is sustainable financing. This study assesses the willingness-to-pay, an integral attribute of sustainable financing mechanism for improved solid waste management (SWM) services in Ghana.

**Methods:**

A cross-sectional multicenter study was conducted in Ghana from January to August 2017 among 1560 households in four regional capitals in Ghana; Accra, Takoradi, Kumasi, and Tamale. Data were collected by using a structured interview questionnaire. Logistic regression models were used to determine the predictors of willingness-to-pay for SWM services in Ghana.

**Results:**

Overall, 53.7% of the households were willing to make additional payment for improved services, with difference across study sites: 54.5%, 53.1%, 61.7%, and 46.6% in Takoradi, Tamale, Accra, and Kumasi, respectively. Willingness-to-pay for improved SWM was predicted by educational level, marital status, type of employment, and region of residence. Compared to women who had no formal education, those having senior high school (aOR (adjusted odds ratio): 2.53; 95% CI: 1.48, 4.30), postsecondary (aOR: 1.97; 95% CI: 1.08, 3.60), and tertiary education (aOR: 3.30; 95% CI: 1.91, 5.69) were associated with higher odds of willingness-to-pay for improved SWM services.

**Conclusion:**

This study provides important evidence on important attribute of financing mechanism, willingness-to-pay for improved SWM services. Findings would contribute to efforts at finding sustainable financing mechanism for waste management services in Ghana.

## 1. Introduction

Solid waste management (SWM) is a major challenge in many urban cities globally, especially developing countries including Ghana. Currently, more than 2 billion people are lacking access to SWM service [1]. In West Africa, the issue of collection, management, and disposal of solid waste continue to feature prominently in major towns and cities as a result of the increasing solid waste, the rising cost of waste management, and the associated environmental and health problems [2].

The World Bank in its quest to achieve eradication of severe poverty and maximizing shared property has spent around 1.2 M dollars in investments and over 55 advisory and analytical works on solid waste programmes and portfolios on about 114 sustainable and active projects within 58 countries [3]. Notwithstanding these interventions, developing countries have seen widening gaps SWM. Ghana currently produces about 13,000 tons of waste daily with over 4,000 tonnes produced in Accra and Kumasi [4, 5]. City authorities and waste management departments are still grappling with how best to deal with this challenge.

In the wake of the SWM challenges, the Government of Ghana revised the Sanitation Policy in 2010 to address the limitations of the old policy published in 1999 and a result of nation-wide consultation among sector stakeholders [6]. The new policy lays the basis for developing a systematic approach and framework for identifying and harnessing resources for value-for-money services to all [6]. The broad principles underlying the revised policy are the principle of environmental sanitation services as a public good; environmental sanitation services as an economic good; the polluter-pays-principle; cost recovery to ensure value-for-money ensuring economy, effectiveness, and efficiency; subsidiarity in order to ensure participatory decision-making at the lowest appropriate level in society; improving equity and gender sensitivity; recognizing indigenous knowledge, diversity of religious and cultural practices; precautionary principle that seeks to minimize activities that have the potential to negatively affect the integrity of all environmental resources; community participation and social intermediation [6]. The focus areas of the policy are capacity development, information, education and communication, legislation and regulation, levels of service, sustainable financing and cost recovery, research and development, and monitoring and evaluation [6].

Privatization of waste collection is suggested as a way to effectively deal with this menace and significantly contribute to improving the environmental sanitation situation in our cities [4]. The involvement of the private sector in SWM has brought some relief to governments; yet, there is still much to be done. In most industrialized cities, SWM service have been provided by private enterprise for decades, and failure of municipal and metropolitan authorities in low- and middle-income countries (LMIC) to keep up with rapid growing cities have necessitated the involvement of the private sector [7]. In Ghana, although private waste management companies have been involved in SWM for some time, the problem of SWM is far from being resolved. Waste management services are still inadequate especially in low-income areas [8] due to inadequate financing, lax attitude of officials and residents, lack of clearly defined roles for stakeholders, poor cost recovery, and institutional weaknesses [9].

Most private waste management companies are privately financed and operate through cost recovery by directly entering into contracts with households for waste management services. The success and financial sustainability of waste management companies therefore relies to some extent on their ability to collect revenue as well as clients' willingness-to-pay for SWM services. It is generally believed that if the households pay more, waste management companies or service providers could increase, review, and improve service delivery. Although some individual studies have attempted to look into this, there has been no nationwide multicenter study in this regard. Thus, this study was conducted to assess the financing options and willingness-to-pay for improved SWM services in Ghana.

## 2. Methods

### 2.1. Study Design and Setting

This was a cross-sectional multicenter study conducted in Ghana from January to August 2017. Geographically, the study area covered four urban centers that are regional capitals in Ghana; Greater Accra (Accra), Ashanti (Kumasi), Western (Takoradi), and Northern (Tamale) coinciding with the three epidemiological zones of northern, middle, and coastal belts. These cities were strategically chosen due to increasing urbanization and high levels of economic activities and accompanied high municipal solid waste generation. These cities have a combined population of 4,566,450 as per the 2010 Ghana Housing and Population Census [10], and they represent the first top four cities by population in Ghana [11]. The vegetation of these cities varies from moist semi-deciduous forest in Kumasi to strand and mangrove zone in Accra to coastal scrubs at Takoradi and savannah grassland in Tamale.

### 2.2. Study Population and Sampling

The study involved heads of households resident in the study area for the past 6 months and consented to participate in the study. The sample size for all the surveys was estimated using the single proportion formula by Cochran [12]; *n* = *z*^2^*pq*/*d*^2^, where *n* = the desired sample size; *z* = the standard normal deviation 1.96 which corresponds to 95% confidence level; *p* = proportion of the target population estimated to use SWM services, set at 60% (0.6); *q*=1 − *P* (1 – 0.6); *d* = margin of error, set at 3%; and *n* = (1.96)^2^ ∗ 0.6 (1 – 0.6)/(0.03)^2^ = 1,024. With a design effect of 1.5, a sample size of 1,536 was rounded up to 1,600 to account for sampling variability.

The sample size per study site was estimated based on the population size in the four study sites as at 2017 [11]. The current population of Ghana is estimated to be 29.46 million, up from 24.2 million in 2010 [11]. Accra is the capital and the largest city of Ghana with a population of 2.27 million, and the Accra Metropolitan area is the largest metro area with about 4 million inhabitants. Kumasi is the second largest city with about 1.5 million inhabitants, and Tamale comes third with a population of about 361 thousand. Takoradi comes fourth with a population of about 233 thousand inhabitants [11]. Based on the population size in the four study sites in 2017, the sample size was allocated as 500 each in Accra and Kumasi and 300 each in Takoradi and Tamale. However, due to errors and inconsistencies, only data from 1560 households were valid and used for the analysis.

The sampling methods combined cluster sampling and simple random sampling techniques. The metropolises were clustered into submetros. Then, within the submetro, the households were selected from clustered households based on electoral areas by random sampling. Those who consented were interviewed, and where there are more than one eligible household, the lottery method was used to pick one household to reduce redundancy. During this exercise, papers with inscription “YES” and “NO” were developed and put in a cup for potential heads of households who met the inclusion criteria to pick. Those who picked “YES” were enrolled while those who picked “NO” were excluded. In each submetro, a household was randomly selected as the starting point. The number of households for the various submetros was calculated with an assumption that 70% of the populations utilized waste management service. All public structures such as schools, hospital, clinic, maternity homes, restaurants, police and military barracks, and prayer camps were excluded.

### 2.3. Data Collection Tools and Techniques

Data were collected using structured questionnaire and checklists comprising both closed and open-ended questions. The questionnaires were administered with assistance of trained graduate research assistants through face-to-face interview. For reliability and validity of the study conclusions, a 3-day training session was held for the research assistants by the principal investigator. Prior to the data collection, the data collection tools were pretested on 50 households. Then, the study was explained to respondents and consent was duly sought. All study protocols were reviewed and approved by the Institutional Review Board of the Kwame Nkrumah University of Science and Technology, Committee for Human Research Publications and Ethics (CHRPE), and informed consent was sought from all participants.

### 2.4. Data Analysis

General characteristics are summarized as proportions and mean ± standard error of the mean (SEM) and stratified by study site. Odds ratios (ORs) and 95% confidence intervals (CIs) were calculated to assess the predictors of willingness-to-pay for SWM services. All statistical tests were conducted at a significance level of *p* < 0.05. Data were analyzed with SPSS version 22 [13].

#### 2.4.1. Estimation Model

The willingness-to-pay for waste management services was estimated based on the contingent valuation (CV) method. Contingent valuation surveys are argued to be a viable method of collecting information on preferences for providing public goods and services in developing countries [12]. Using this method, many empirical studies have provided evidence that households are willing to pay a significant amount for the provision of improved waste management [14–16].

The willingness-to-pay by a group of consumers for a particular product at a price (or bid) *B* can be assumed to have a certain probability distribution function [17]. This distribution function can be seen as a function of price, with a higher price having lesser probability of being accepted. In applied research, the logistic distribution is commonly used, and the effect of price is entered indirectly in an argument called the index function, denoted as *v*. The most common index function is linear in the price or bid *B:*

(1) $$\begin{array}{c} v=\alpha -\rho B, \end{array}$$

and the probability distribution of the WTP is then presented by

(2) $$\begin{array}{c} P\left(\text{WTP}=B\right)=\frac{\exp\left(v\right)}{{\left(1+\exp\left(v\right)\right)}^{2}}. \end{array}$$

The logistic function has the advantage of a closed-form cumulative distribution function *G*(.), which then represents the proportion of the population whose WTP lies below a certain value *B*:

(3) $$\begin{array}{c} G\left(B\right)=P\left(\text{WTP}<B\right)=\frac{\exp\left(v\right)}{\left(1+\exp\left(v\right)\right)}. \end{array}$$

People who will accept an offer of value *B* are those whose WTP is equal to or higher than *B*.

In the double-bounded dichotomous choice model, the consumer is presented with two consecutive bids, and the second bid depends on the response to the first. If the consumer answers “yes” to the first bid (*B*), the second bid (*B*^*u*^) is set *i* higher, but if the individual responds “no” to the first bid, the second bid (*B*^*d*^) is set lower. There are four possible outcomes: “yes/yes” to the first bid followed by a “yes/no”, “no/yes”, and “no/no”, denoted by (*π ^yy^* ), (*π ^yn^* ), (*π ^ny^* ), and (*π ^nn^* ), respectively.

To receive information on a wider range of values, different amounts for the bids are assigned randomly between respondents *i*. The probability of receiving a “yes” answer to both questions equals to the probability that the respondent's WTP is higher than the highest bid offered:

(4) $$\begin{array}{c} \pi_{i}^{yy}\left(B_{i},B^{u}\right)={\mathrm{Pr}}_{i}\left(B^{u}<{\text{WTP}}_{i}\right)=1-G_{i}\left(B^{u}\right). \end{array}$$

Similarly, the probability of receiving a “yes” followed by a “no” equals the probability that the WTP of respondent *i* lies between the initial bid and the second bid, higher bid offered:

(5) $$\begin{array}{c} \pi_{i}^{yn}\left(B_{i},\;B^{u}\right)={\mathrm{Pr}}_{i}\left(B_{i}^{u}<{\text{WTP}}_{i}<B^{u}\right)=G\left(B^{u}\right)-G\left(B\right). \end{array}$$

The probability of receiving a “no” followed by a “yes” is again the probability that WTP lies between the individual and the second bid, now the lower bid offered:

(6) $$\begin{array}{c} \pi_{i}^{ny}\left(B_{i},B^{d}\right)={\mathrm{Pr}}_{i}\left(B^{d}<{\text{WTP}}_{i}<B\right)=G\left(B\right)-G\left(B^{d}\right). \end{array}$$

Finally, the probability of receiving two “no” answers are equal to the probability that WTP lies below the second bid offered:

(7) $$\begin{array}{c} \pi^{nm\;\;}\left(B,\;\;B^{a}\right)=\mathrm{Pr}\left(B^{a}<\text{WTP}\right)=G\left(B^{a}\right). \end{array}$$

Combining the probabilities of four outcomes, the log-likelihood function for a sample of *N* consumers takes the following form:

(8) $$\begin{array}{c} \ln L^{\text{D}}\left(\theta \right)=\overset{N}{\underset{i=1}{\sum }}\left\{d_{i}^{yy}\ln\pi^{yy}\left(B_{i},B_{i}^{u}\right)+d_{i}^{nm}\ln\;\pi^{nm\;\;}\left(B_{i},B_{I}^{d}\right)+\;\;d_{i}^{yn}\ln\;\pi^{yn\;\;}\left(B_{i},B_{I}^{d}\right)+\;\;d_{i}^{ny}\ln\;\pi^{ny\;\;}\left(B_{i},B_{I}^{d}\right)\right\},\;\; \end{array}$$

where *d*_*i*_^*yy*^, *d*_*i*_^*mn*^, *d*_*i*_^*yn*^, and  *d*_*i*_^*ny*^ are binary variables with 1 denoting the occurrence of that particular outcome and 0 otherwise.

Household willingness-to-pay for improved SWM can be specified as follows:

(9) $$\begin{array}{c} \text{WTP}=\;\;\alpha +\;\;\rho b+\;\;\beta z+\;\;\varepsilon , \end{array}$$

where *b* represents the last bid level which the respondent was offered, *z* is the socioeconomic factor, and *ε* is the random variable accounting for unobserved factors, and *α*, *ρ*, and  *β* are parameters to be estimated.

The empirical formulation of equation (9) is finally formulated as follows:

(10) $$\begin{array}{c} \text{WTP}=\;\;\alpha +\;\;\rho b+\;\;\beta_{1}\text{AGE}+\beta_{2}\text{GEN}+\beta_{3}\text{EDU}+\beta_{4}\text{MARI}+\beta_{5}\text{EMP}+\beta_{6}\text{OCC}+\beta_{7}\text{INC}+\beta_{8}\text{RESI}+\;\;\varepsilon , \end{array}$$

where AGE is the age of the respondent, GEN is the gender of the respondent, EDU is the level of education of the respondent, INC is the average monthly income of the respondent, MARI is the marital status of the respondent, EMP is the employment status of the respondent, OCC is the occupation of respondent, and RESI is the residence of the respondent. A description of the explanatory variables is provided in Table 1.

## 3. Results

### 3.1. Background Characteristics of Respondents

The mean age ± SEM of the study participants was 35 ± 0.3 years, and participants from the Northern and Ashanti regions were significantly older than those from the Western and Accra (Table 2). Overall, less than half (45.8%) of the study participants were men. The educational level was highest in Accra and lowest in Kumasi where there was lowest proportion of participants with tertiary education (25.5%) and highest proportion of respondents with no formal education (15.0%). Most of the participants were employed, and Kumasi had the highest proportion of participants working in the informal sector. The proportion of residents in estates and new sites was highest in Takoradi and lowest in Kumasi, whiles the Kumasi site had the highest proportion of participants resident in old towns.

### 3.2. Financing Options and Willingness-to-Pay for Improved Solid Waste Management Services

**Figure 1:**
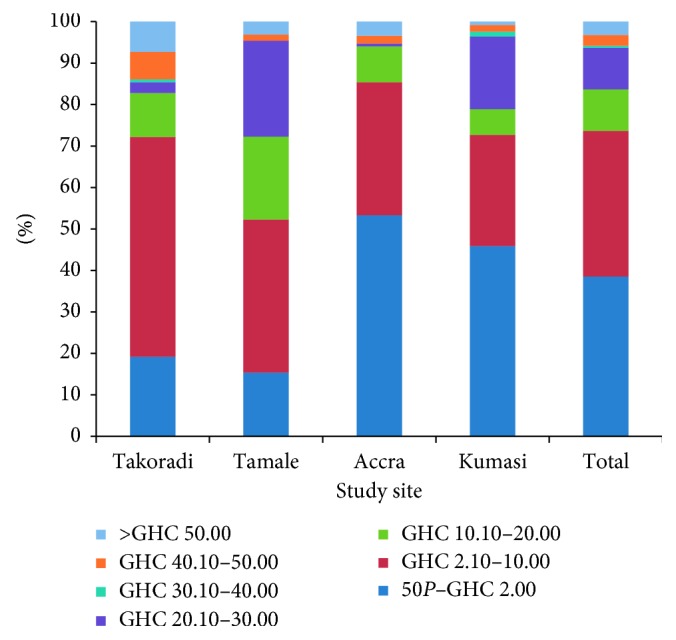
Additional amount households are willing to pay on top of the current price.

The current financing options for most of the households were user charges and self-payments (Table 3). Majority of the households believe that the current payment options were effective, whereas others believe the payment systems have effect on service delivery. Some of these effects, as cited by respondents, include dumping of refuse elsewhere (2%), discouraging continuous use of services (65%), poor service delivery (32%), and arbitrary charging (1%). Generally, majority were willing to make additional payment for improved quality of services, with increased frequency of lifting being the most cited motivation for paying extra. Majority of households across the various sites were willing to pay from 50p to GH¢10.00 in the overall population as additional fee for improved services (Figure 1). Across the various sites, majority were willing to pay up to GH¢2.00 in Accra and Kumasi, whereas majority from Tamale and Takoradi were willing to pay from GH¢2.00 to GH¢10.00. Reasons for not paying extra included taxation, low income and economic reasons, collection frequency not adhered to by waste management companies, the belief that Government should pay, and inadequate/poor service delivery.

Table 4 presents results of the univariable and multivariable logistic regression analysis of socioeconomic predictors of willingness-to-pay for improved SWM services. The multivariable analysis indicated that willingness-to-pay for SWM services could be predicted by educational level, marital status, socioeconomic status, area of occupation, and region of households. In the univariable analysis, having senior high school and tertiary level education was associated with higher odds of willingness-to-pay for the management of solid waste. In the adjusted model, having any form of education was significantly associated with higher odds of willingness-to-pay for SWM compared to those who had no form of education. As compared to civil servants, being self-employed was associated with 44% lower odds of willingness-to-pay for improved SWM services (aOR: 0.56; 95% CI: 0.40, 0.81).

## 4. Discussion

Poor waste management services is a global challenge compounded by clients' unwillingness to pay, especially in developing countries like Ghana, where a majority of people live on less than a dollar a day making central government more vulnerable to absorb cost of service. In contrast, in developed countries, the involvement of the private sector has contributed immensely to effective waste management. A key challenge of waste management in developing countries is sustainable financing mechanism [18, 19]. Currently, the most available payment option for SWM is polluter or user pay. Waste management companies however do not realize enough from user payments, to be able to provide quality services owing to inability to collect all revenues from households and households' inability or unwillingness to pay for SWM services.

In this study, most households were willing to make additional payment for improved quality of SWM services, preferably increase in the frequency of lifting of garbage. Reasons for not willing to pay extra included low income and economic reasons, collection frequency not adhered to by waste management companies, the belief that Government should pay, and inadequate/poor service delivery. This corroborates findings from a study in the Kumasi metropolis where majority were willing to pay additional fees for improved service [20]. Similarly, their study reported some respondents' unwillingness to pay because they believe it is the responsibility of government to pay for cost related to waste management services.

Most of the households were willing to pay up to GH¢10.00 in addition for improved services. Similarly, in the study by Awunyor-Vitor et al. [20] in the Kumasi metropolis 5 years ago, households were willing to pay at least GH¢ 5.00 or more to support improved waste management services. This suggests that, presently, an increment of about GH¢5.00–GH¢10.00 will be acceptable to majority of households if it is accompanied by improved services by waste management companies.

Currently, self-payments and user charging are the prevailing financing options for household waste collection in Ghana. In Europe, the polluter pay principle has been part of waste management legislation since 1975 [21]. This payment system is readily understood by users and in line with the “polluter pays principle” (users producing more or more hazardous/nonrecyclable waste have to pay more) [22]. This type of user charge system could serve not only as a cost recovery instrument but also as a motivation to reduce the production of waste [22].

Some households in this study believed this payment option has effects on service delivery by encouraging indiscriminate dumping of waste, discouraging continuous payment, and promoting arbitrary charging and poor service delivery. A setback of this payment system however is the inability to collect substantial revenue to cover their cost of operations due to poor payment mechanism or mode of payments. In a study of financial sustainability of SWM in Ethiopia [23], the efficiency of fee collection from households was only around 50%, with the total amount of revenues insufficient to cover the running costs of waste management. Their study suggested a detailed cost structure and cost-revenue analysis of waste management activities in order to enhance cost efficiency and balance the cost revenues towards cost recovery. It is believed that with an effective revenue collection mechanism in place, the user charge or polluter pay system will be an effective financing option for SWM in Ghana and similar settings. We suggest education of the populace on SWM and utilization of electronic platforms for payment of user fees as a way of improving revenue mobilization under this payment system. Public education has been found to be a useful tool in promoting positive waste management behaviour among households [24, 25].

### 4.1. Predictors of Willingness-to-Pay for Improved Solid Waste Management in Ghana

Educational level, marital status, area of occupation, and region of residence predicted willingness-to-pay extra for improved SWM services in Ghana. Having any form of education was significantly associated with higher odds of willingness-to-pay for SWM compared to those who had no form of education. Findings from this study corroborate previous studies from Ghana and other settings, which found an association between educational level and willingness-to-pay for improved SWM services [26–28]. The highly educated have higher income and improved socioeconomic status [29] and are therefore more likely to pay and utilize SWM services. Besides being economically empowered, education leads to positive perceptions and ability to understand the health implications of indiscriminate waste disposal [30–32]. In Ghana, low education is believed to contribute to poor waste management practices [31], and high education is positively linked to the willingness-to-pay for improved SWM services [20].

Previous studies have shown that the use of SWM services is dependent on the ability to pay for the services [20, 26]. Households with high-income level would therefore be more willing to use SWM services and to pay for improved services. In this study, household heads who rated themselves poor had lower odds of willing to pay extra for improved SWM services. Similarly, studies conducted in Malaysia [33] and Sri Lanka [34] reported an influence of household income on willingness-to-pay for improved SWM services. A similar study conducted among randomly selected households in Ethiopia also revealed that improved SWM is significantly related to income [35].

### 4.2. Strengths and Limitations

This study is the first nationwide survey that looks at the current financing options and the willingness-to-pay for improved SWM services in Ghana. By ensuring consistent measures and data collection across sites, we believe findings from this study are more generalizable and applicable to the Ghanaian context. This study also documented important predictors of utilization of SWM in Ghana; nonetheless, we could not explore all of these factors. Household wealth, for instance, was not assessed and utilized in this study. We however assessed the perception of socioeconomic status, income, and educational levels, which we believe are useful proxies for the socioeconomic position of the household.

## 5. Conclusion

In conclusion, slightly over half of households (53.7%) in Ghana are willing to pay extra for improved SWM services in Ghana. Educational level, marital status, region of residence, and rate of socioeconomic status predict the willingness-to-pay for improved SWM services in Ghana. The current payment option for SWM is user fees, and most households believe that this discourages the continuous use of SWM services. An understanding of these issues is of practical importance and provides relevant considerations to help improve SWM services in the country. The operational challenges of SWM companies, municipal, and metropolitan assemblies are important areas for further research and would help understand and address the problem of SWM more holistically.

## Figures and Tables

**Table 1:** Description of explanatory variables.

Variable	Contextual definition
Age	Age of respondents in years
Sex	Sex of respondents: male, female
Education	Educational level of respondents: no formal, basic, senior high school, postsecondary, tertiary
Marital status	Married, cohabiting, single, divorced, or widowed
Employment status	Respondents' employment status: employed, unemployed
Area of occupation	Private sector, government (civil or public servants), self-employment (informal sector)
Residence	Urban, periurban, or rural
Description of residency	Estate, new site, inner-city or old town, slums, zongos
Rate of economic status	Very rich, rich, moderately rich, poor, very poor
Duration of stay in community	Length of stay of respondents in their respective communities
Amount earned from economic activities	Total monthly income from all economic activities
Study site	Cities where the study was conducted

**Table 2:** Socioeconomic characteristics of respondents, stratified by study site.

Variables *N*	Total (*N*=1560)	Takoradi (*N*=295)	Tamale (*N*=297)	Accra (*N*=467)	Kumasi (*N*=501)
Age in years, mean ± SEM	35 ± 0.3	29 ± 0.5	38 ± 0.7	31 ± 0.4	39 ± 0.6
Gender household head, male, (%)	45.8	51.2	28.6	60.0	39.7
Education, *N* (%)					
Never been to school	7.50	2.40	10.4	0.90	15.0
Basic education^*∗*^	11.8	10.2	12.5	1.7	21.8
Senior high school	20.7	17.3	18.2	14.6	29.9
Postsecondary (training/vocational)	9.00	13.2	12.8	5.10	7.80
Tertiary (diploma and degree)/others	51.0	56.9	46.1	77.7	25.5
Marital status, (%)					
Single	38.8	62.7	18.5	48.8	27.3
Married	53.3	32.9	73.7	43.5	62.3
Cohabitation	2.90	2.00	1.00	6.00	1.80
Widow/divorce	5.00	2.40	6.70	1.70	8.60
Employed (%)	93.5	91.9	93.8	91.4	96.2
Occupation (%)					
Government (civil servants)	19.4	32.5	29.6	16.1	8.6
Private	31.3	26.4	17.8	57.2	18.2
Self-employment (informal sector)	49.3	41.0	52.5	26.8	73.3
Type of occupation (%)	*N*=1347	*N*=260	*N*=272	*N*=396	*N*=419
Skilled	62.1	60.4	66.2	75.8	47.5
Semiskilled	27.2	30.0	18.8	17.9	39.9
Unskilled	10.7	9.60	15.1	6.30	12.6
Amount earned from all economic activities, GH¢, median (25th and 75th percentiles)	*N*=1218 900.00 (500.00, 1500.00)	*N*=227 800.00 (500.00, 1500.00)	*N*=228 1000.00 (500.00, 1600.00)	*N*=323 1100.00 (600.00, 2000.00)	*N*=440 800.00 (500.00, 1200.00)
≤300	12.3	12.3	10.1	7.7	16.8
300–600	22.7	30.0	21.9	18.0	23.0
600–100	27.4	25.1	24.6	23.8	32.7
>1000	37.5	32.6	43.4	50.5	27.5
Rate of socioeconomic status					
Very rich	6.5	9.7	2.4	3.2	10.2
Rich	11.0	12.0	13.4	6.4	13.4
Moderately rich	73.0	66.7	66.4	84.6	69.9
Poor	9.40	11.7	17.8	5.80	6.60
Place of residence (%)					
Estate	18.8	27.8	13.5	27.2	8.8
New site	27.1	36.6	26.9	26.3	22.2
Inner-city	16.8	10.2	12.5	28.7	12.2
Old town	23.5	11.5	25.9	7.9	43.7
Slums	1.20	1.70	1.70	0.90	0.80
Zongos	7.90	8.80	15.80	4.90	5.40
Others	4.80	3.40	3.70	4.10	7.00
Description of residency (%)					
Urban	76.7	76.6	79.1	76.9	75.0
Periurban	23.3	23.4	20.9	23.1	25.0
Duration of stay in community, years, median (25th and 75th percentiles)	*N*=1467 7 (4, 17)	*N*=291 5 (3, 11)	*N*=260 6 (3, 18)	*N*=445 7 (4, 15)	*N*=471 10 (4, 20)
<5	41.2	54.0	50.0	40.2	29.3
5–10	24.5	21.0	21.2	27.6	25.5
10–15	8.20	6.90	3.10	10.1	10.2
15–20	10.2	13.7	4.60	8.50	12.5
>20	16.0	4.5	21.2	13.5	22.5
Number of dependents median (25th and 75th percentiles)	*N*=1223 2 (3,5)	*N*=119 2 (2, 4)	*N*=76 4 (3, 6)	*N*=155 3 (2, 4)	*N*=313 3 (2, 5)
0	1.20	0.00	0.00	2.20	1.70
1	15.1	20.4	6.4	15.9	16.7
2	24.8	40.7	15.7	25.1	20.9
3	17.7	12.4	19.1	22.8	15.2
≥4	41.2	26.5	58.9	34.0	45.5

SEM, standard error of the mean; ^*∗*^primary and junior high school.

**Table 3:** Willingness-to-pay for waste management services.

Variables *N*	Total (*N*=1560) (%)	Takoradi (*N*=295) (%)	Tamale (*N*=297) (%)	Accra (*N*=467) (%)	Kumasi (*N*=501) (%)
Current financing option for your household waste					
User charges	43.1	55.7	32.5	33.5	48.8
Through taxes	5.30	4.40	4.80	3.50	7.50
Subsidy from assembly	4.80	6.20	10.0	4.30	1.80
Government grants	4.50	2.60	3.00	0.80	9.50
Self-payments	42.3	31.1	49.8	57.9	32.5
Current financing option effective	76.2	61.9	68.9	74.7	89.6
Current financing option have effect on the service delivery	69.6	61.0	74.7	55.0	84.7
Worth paying for the services of waste collection/management	82.0	77.8	78.8	89.7	78.9
Method of pay for existing services					
Revenue collector	64.2	74.9	75.1	81.9	27.6
Pay point outlet	24.5	15.5	17.2	9.60	53.6
Walk to service provider's office	10.2	7.30	7.70	7.00	18.1
Through bank	0.40	1.80	0.00	0.00	0.00
Others	0.70	0.50	0.00	1.5.0	0.70
Satisfied with the mode of payment	82.7	74.1	82.8	81.6	88.7
If no, preferred payment model					
Add to electricity bill	29.7	8.00	75.0	26.1	6.90
Add to water bill	12.9	13.0	11.3	16.8	10.7
Add to property rate	20.9	34.0	3.2	29.4	19.8
Taxation	34.8	43.0	8.1	25.2	62.6
Others	1.70	2.00	2.40	2.50	0.00
Consider waste management as a public good	82.8	86.3	86.8	89.9	72.2
Who you think should pay for your waste collection					
Local government/assembly	32.7	55.3	29.1	34.8	34.4
Community	3.40	1.70	2.20	10.6	5.20
Taxation	12.0	9.6	14.0	13.8	12.8
Self-payment	51.9	32.1	53.7	40.6	45.1
Others	0.00	1.30	0.90	0.20	2.40
Prepared to pay something towards the treatment and disposal of waste	62.2	57.7	56.5	67.1	64.1
If no, whom you think should pay for treatment and disposal					
Municipal assembly	61.2	64.2	75.7	63.2	49.0
Service provider	13.7	16.8	2.8	15.8	16.5
Through tax	24.1	17.9	19.4	20.0	34.6
Others (most cited: government)	0.80	1.20	2.10	1.00	0.00
Willing to make additional payments for improved quality of services. Motivation to pay extra	53.7	54.5	53.1	61.7	46.6
Increase in frequency of lifting	62.5	50.5	70.3	51.6	83.4
Adding bin liner	13.6	11.5	14.1	20.1	7.2
Effective communication	11.5	20.3	12.5	8.4	7.6
Provision of free receptacle	10.8	17.6	3.1	15.4	1.8
Others	1.60	0.00	0.00	4.40	0.00

**Table 4:** Logistic regression analysis of socioeconomic predictors of willingness-to-pay for solid waste management services.

Variables	Crude OR (95% CI)	Adjusted OR (95% CI)
Age in years	1.01 (1.00, 1.02)^∗^	**—**
Male gender		
Male	0.96 (0.79, 1.17)	**—**
Female	1.00	**—**
Educational level		
No formal education	1.00	1.00
Basic education	1.53 (0.96, 2.46)	1.76 (1.01, 3.06)^∗^
Senior high school	1.94 (1.26., 2.30)^∗^^∗^	2.52 (1.48, 4.30)^∗^^∗^
Postsecondary	1.51 (0.92, 2.49)	1.97 (1.08, 3.60)^∗^
Tertiary	2.19 (1.47, 3.26)^∗^^∗^^∗^	3.30 (1.91, 5.69)^∗^^∗^^∗^
Marital status		
Married	1.00	1.00
Cohabiting	1.20 (0.97, 1.47)	1.47 (1.13, 1.93)^∗^^∗^
Single	1.01 (0.56, 1.84)	3.61 (1.53, 8.52)^∗^^∗^
Widowed/divorced	0.77 (0.42, 1.43)	0.32 (0.12, 0.81)^∗^
Employed		
Yes	1.63 (1.09, 2.45)^∗^	**—**
No	1.00	**—**
Area of occupation		
Government (civil servants)	1.00	1.00
Private	0.81 (0.60, 1.08)	0.56 (0.40, 0.81)^∗^^∗^
Self-employment (informal sector)	0.80 (0.61, 1.05)	0.75 (0.52, 1.08)
Description of income level		
Less than 500 GHC	1.00	1.00
500–1000 GHC	1.30 (0.95, 1.77)	1.15 (0.81, 1.64)
1100–2000	1.04 (0.76, 1.41)	0.72 (0.51, 1.04)
2100–3000	0.92 (0.59, 1.45)	0.70 (0.42, 1.15)
3100–5000	1.62 (1.02, 2.55)^∗^	1.07 (0.63, 1.83)
Rate of socioeconomic status		
Very rich	1.00	1.00
Rich	1.71 (1.00, 1.29)^∗^	1.57 (0.84, 2.95)
Moderately rich	0.63 (0.41, 0.94)^∗^	0.64 (0.38, 1.08)
Poor	0.31 (0.18, 0.53)^∗∗∗^	0.28 (0.14, 0.51)^∗∗∗^
Residence		
Urban	1.26 (1.00, 1.59)^∗^	**—**
Periurban	1.00	**—**
Description of residency		
Estate	1.00	1.00
New site	0.85 (0.63, 1.15)	0.94 (0.66, 1.34)
Inner city/old town	0.90 (0.68, 1.19)	1.22 (0.85, 1.74)
Slums/zongos/others	0.55 (0.37, 0.92)^∗∗^	0.74 (0.46, 1.20)
Region		
Western	1.00	1.00
Northern	0.82 (0.59, 1.13)	1.05 (0.69, 1.58)
Greater accra	0.99 (0.74, 1.33)	1.04 (0.72, 1.50)
Ashanti	1.32 (0.99, 1.76)	1.52 (1.04,2.22)^∗^

SWM, solid waste management. ^∗^p < 0.05; ^∗∗^p < 0.01; ^∗∗∗^p < 0.001.

## Data Availability

Data for this study are securely kept by the principal investigator and will be made available upon request.
